# Defining the PTSD Service Dog Intervention: Perceived Importance, Usage, and Symptom Specificity of Psychiatric Service Dogs for Military Veterans

**DOI:** 10.3389/fpsyg.2020.01638

**Published:** 2020-07-21

**Authors:** Kerri E. Rodriguez, Megan R. LaFollette, Karin Hediger, Niwako Ogata, Marguerite E. O’Haire

**Affiliations:** ^1^Center for the Human-Animal Bond, Purdue University, West Lafayette, IN, United States; ^2^Department of Comparative Pathobiology, Purdue University, West Lafayette, IN, United States; ^3^Department of Animal Sciences, College of Agriculture, Purdue University, West Lafayette, IN, United States; ^4^Department of Clinical Psychology and Psychotherapy, University of Basel, Basel, Switzerland; ^5^Department of Veterinary Clinical Sciences, College of Veterinary Medicine, Purdue University, West Lafayette, IN, United States

**Keywords:** PTSD service dogs, psychiatric service dogs, military veterans, PTSD, animal-assisted intervention, human–animal interaction, human–animal bond

## Abstract

Research suggests that psychiatric service dogs may be an effective complementary treatment option for military veterans with posttraumatic stress disorder (PTSD). Although this practice continues to increase in popularity and research has reached the rigor of clinical trials, the components of the PTSD service dog intervention remain largely undefined. This research aimed to (1) quantify the importance, usage, and PTSD symptom specificity of service dog trained and untrained behaviors, (2) explore how PTSD severity, time since receiving the service dog, and the veteran-dog relationship relate to outcomes, and (3) compare expectations of veterans on the waitlist to experiences of veterans with service dogs. In a cross-sectional design, 217 post-9/11 military veterans with PTSD were recruited from a national service dog provider, including *n* = 134 with a service dog and *n* = 83 on the waitlist. Results showed that the service dog’s trained tasks of calming and interrupting anxiety were perceived as the most important for veterans’ PTSD, the most frequently used in a typical day, and as helping the most PTSD symptoms. Trained tasks were most helpful to the PTSD symptoms of hypervigilance and intrusion, and least helpful toward the symptoms of amnesia and risk-taking. Although all trained tasks were helpful toward PTSD symptoms, veterans rated the service dog’s untrained behaviors on average as more important for their PTSD. After controlling for covariates, there was no relationship between a veteran’s PTSD severity and perceived importance or frequency of task use. However, veterans who reported feeling closer to their service dogs reported using trained tasks more often, and veterans who had their service dogs for longer reported using trained tasks less often. Finally, veterans on the waitlist reported higher expectations regarding task use and importance than described by veterans with a service dog. In conclusion, findings describe the core components of the PTSD service dog intervention by quantifying the use and value of trained and untrained dog behaviors. Overall, this study helps explain the PTSD service dog’s clinically relevant value while contributing to the scientific understanding of this emerging practice.

## Introduction

Of the roughly 2.7 million United States military personnel deployed to Iraq and Afghanistan post-9/11, up to 23% return with diagnostic symptoms of posttraumatic stress disorder (PTSD; Fulton et al., 2015). PTSD is a pervasive mental health condition that can occur after exposure to a traumatic event characterized by avoidance, re-experiencing, negative alterations in cognition and mood, and hyperarousal (American Psychiatric Association, 2013). Several evidence-based treatment options for PTSD exist, including cognitive behavioral therapy, prolonged exposure therapy, and pharmacotherapy (Foa et al., 2008). However, treatment dropout rates are often high among military veterans, and many veterans will retain their PTSD diagnosis despite treatment completion (Resick et al., 2015; Steenkamp et al., 2015). To meet the needs of military veterans with pervasive PTSD symptoms, many complementary and alternative treatments and practices have emerged to supplement evidence-based care (McPherson and Schwenka, 2004).

One increasingly popular integrative treatment option for PTSD is the provision of a specially trained psychiatric service dog. Psychiatric service dogs are a form of assistance dog that are specially trained to do work or perform tasks directly related to a psychiatric disability – thereby allowing them legal public access rights (Americans with Disabilities Act of 1990). For example, PTSD service dogs can be trained to detect a veteran’s physical signs of anxiety and distress, serving to alert to and interrupt anxiety and panic attacks during the day as well as interrupt nightmares during the night. PTSD service dogs can also be trained for positional commands thought to provide a sense of safety in public, such as standing behind the veteran in public and “watching their back.” The resulting companionship and non-judgmental social support that a PTSD service dog provides can also offer emotional and therapeutic value (Krause-Parello and Morales, 2018). PTSD service dogs are referred to as an complementary intervention as this practice is considered a non-traditional approach to supplement evidence-based care and mainstream therapies (O’Haire and Rodriguez, 2018; Scotland-Coogan et al., 2020). The demand for PTSD service dogs continues to increase, waitlists for PTSD service dogs are often months or years long (Walther et al., 2017, 2019). PTSD service dogs may be popular due to the low perceived stigma surrounding this practice in comparison to other forms of mental health treatment (Kim et al., 2010; Yarborough et al., 2017).

Recent research has provided preliminary evidence of the therapeutic efficacy of PTSD service dogs for military veterans. Cross-sectional studies suggest that compared to receiving usual care while on the waitlist, having a PTSD service dog is associated with lower PTSD symptoms, better quality of life, and better social functioning in addition to more regulated production of the stress hormone cortisol (Yarborough et al., 2017; O’Haire and Rodriguez, 2018; Rodriguez et al., 2018). Similarly, longitudinal studies have found that after receiving a PTSD service dog, veterans self-report significant improvements to PTSD symptoms in addition to secondary outcomes such as depression, anxiety, and quality of life (Kloep, 2016; Bergen-Cico et al., 2018; Whitworth et al., 2019). This emerging literature base is complemented by qualitative reports suggesting that PTSD service dogs can provide significant social and emotional support, reduce stress, and improve veterans’ overall quality of life (Taylor et al., 2013; Yount et al., 2013; Krause-Parello and Morales, 2018).

Despite recent knowledge gained regarding the psychosocial and physiological effects of PTSD service dogs, the therapeutic components of the intervention remain largely undefined. Various proposed standards for PTSD service dog training agree that dogs must be trained for tasks to mitigate the veterans’ PTSD (Assistance Dogs International, 2019). However, these trained tasks not only vary widely across service dog providers, but also according to an individual veteran’s needs (Vincent et al., 2019). There is a critical need for an empirical assessment of the perceived clinically relevant value of specific trained tasks and behaviors for military veterans with PTSD. This information is especially relevant for understanding how these psychiatric service dogs may serve as an complementary treatment option for PTSD. Further, it is unknown how important both untrained and trained behaviors are for managing PTSD symptoms, how often trained tasks are used on a daily basis, and how these outcomes may relate to PTSD symptom severity, time since receiving the service dog, and the human–animal bond. As research in this area reaches the rigor of clinical trials (ClinicalTrials.gov, 2019a, b), such knowledge is crucial to be able to interpret outcomes, understand potential mechanisms of action, and optimize future therapeutic efficacy.

The purpose of this exploratory, non-hypothesis driven study was to define the PTSD service dog intervention by quantifying its therapeutic components utilizing self-reported data from a population of military veterans both with a service dog and on the waitlist to receive one. Specifically, this research aimed to (1) quantify the importance of both trained and untrained service dog behaviors toward veterans’ PTSD (2) describe the frequency of use and PTSD symptom specificity of trained service dog tasks, (3) determine how PTSD symptom severity, the veteran-service dog relationship, and time since the service dog was placed may relate to importance and usage outcomes, and (4) compare the expectations of those on the waitlist to the everyday experiences of veterans with service dogs.

## Materials and Methods

### Participants

Using a cross-sectional design, participants both with a service dog and on the waitlist to receive a service dog were recruited to participate in an online survey. Participants were recruited between January and May of 2016 from the database of the United States service dog provider K9s For Warriors (Ponte Vedra Beach, FL, United States). K9s For Warriors is an Assistance Dogs International (ADI)-accredited, non-profit organization that provides service dogs free of charge to post-9/11 military veterans in almost all 50 U.S. states. Participants consisted of those who applied for and had been approved to receive a PTSD service dog from K9s For Warriors, which utilizes the following inclusion criteria for placements: Verified honorable discharge or current honorable service in the United States armed forces, verification of a service-connected disability, verified diagnosis of PTSD from a clinician referral letter or met the clinical cutoff of 50 on the PTSD Checklist (PCL-IV; Weathers et al., 1993), passed a background check verifying no conviction of any crime against animals or felony convictions, had no current substance abuse, was independently mobile, and had no more than two pet dogs living in the home (per the policies of the service dog provider).

A total of 217 military veterans with PTSD participated in the survey (response rate of 51%), including 134 placed with a service dog and 83 on the waitlist to receive one. Participants *on the waitlist* had been approved to receive a service dog from the provider (i.e., had completed the application and passed screening from the organization) but had not yet received a service dog at the time of participation in the research. The exact length of time on the waitlist was unknown for each participant, but both previous research with this population (O’Haire and Rodriguez, 2018) and reports from the service dog provider indicate that veterans spend an average of 18 months on the waitlist.

Participants *with a service dog* had received a service dog from the provider between 1 month and 7.17 years prior to participating in the research (*M* = 1.80, *SD* = 1.67, Median = 1.33 years). Service dog placement occurred onsite at K9s For Warriors campus during a 3-week class. During this time, groups of 6–10 veterans received daily instruction to learn how to interact with, care for, and continue training their service dogs at home. Service dogs were primarily sourced from shelters and selected based on their age, temperament, and physical size. Specifically, dogs are screened for physical soundness and health, and selected for friendly temperaments, lack of any aggression or fear, and overall trainability. At full maturity, dogs must be at least 24 inches tall and weigh at least 50 pounds to serve as a potential bracing object for veterans needing assistance with balance. Breeds were predominantly Labrador Retrievers or Labrador Mixes. Dogs were trained for a minimum of 120 h before placement on basic obedience (e.g., sit, stay, down, and recall) and specific tasks to mitigate PTSD symptoms (see Table 1 for the list of tasks trained by the organization). Before final placement, veteran-service dog pairs were required to pass a public access certification test to demonstrate appropriate control and service dog behavior in public settings.

**TABLE 1:** Service dog trained behaviors and untrained behaviors or characteristics as described to participants in the survey.

**Trained behaviors**	
Interrupt/alert to anxiety	The dog lets the veteran know when they are feeling anxious and interrupts with a nose bump, placing head in lap, or some other behavior.
Calm/comfort anxiety	The dog performs a calming behavior such as making physical contact (laying on top of handler, placing head in lap, gently leaning against the body) when the veteran feels distress or anxiety.
Block (create space)	The dog positions itself horizontally in front of the veteran to create personal space.
Block (guard/protect)	The dog positions itself horizontally in front of veteran to guard/protect.
Cover (watch back)	Dog positions itself directly behind the veteran to “watch” the veteran’s back.
Social greeting	The dog helps greet people in public by sitting/offering a paw.
Wake up from nightmare	The dog recognizes that the veteran is having a nightmare and gently wakes them up.
**Untrained behaviors or characteristics**	
Companionship	Dog is a “battle buddy,” best friend, and companion.
Non-judgmental	Dog does not judge person for PTSD.
Love	Dog gives person something to love, and to feel loved in return.
Calming	The dog’s physical presence is calming and comforting.
Happiness	Dog makes person smile and brings joy to their life.
Independence	Dog is source of empowerment for veteran to do things on their own.
Leave house	Dog enables veteran to leave house and feel at ease in public.
Connecting to family	Dog helps connect veteran to their family.
Routine	Dog adds structure, routine, and responsibility to veteran’s life.
Social help	Dog helps the veteran make friends and have comfortable social interactions.

Apart from the service dog intervention, neither the service dog provider nor the researchers encouraged or discouraged any treatments or intervention services for participants’ PTSD. Thus, all participants received unrestricted access to usual care for their PTSD symptoms.

### Procedure

The study protocol was approved by the Purdue University Human Research Protection Program Institutional Review Board (IRB Protocol 1607017967). Because there were no interactions between researchers and service dogs, a waiver was obtained by the Purdue University Institutional Animal Care and Use Committee (IACUC). To recruit participants, researchers obtained contact information including veterans’ names and email addresses from the service dog provider. Potential participants were recruited via a personalized email which included information about the study and a link to complete an online survey regarding their experiences and perceptions about PTSD service dogs (dog-specific outcomes including service dog training, temperament, and personality have been published in a separate manuscript; LaFollette et al., 2019). Participants were advised that their individual answers would be kept confidential and would not be shared with the service dog provider. Voluntary informed consent was obtained electronically by asking participants to confirm that they understood the research study and details regarding their participation before clicking “next” on the survey’s landing page. Upon completion of the survey, participants chose between receiving $20 in cash (42%) or $20 Amazon gift card (58%) as compensation for their time.

### Measures

#### Demographics

The online survey contained demographic questions including age, gender identity, marital status, and current pet dog ownership. Participants also consented for researchers to access their records with the service dog provider, which shared service dog placement information (month and year) for those already placed with a service dog.

#### PTSD Symptoms

Posttraumatic stress disorder symptom severity was assessed with the PTSD Checklist (PCL-5) for the Diagnostic and Statistical Manual of Mental Disorders (DSM-5; Blevins et al., 2015). The PCL-5 is a 20-item questionnaire assessing current PTSD symptom severity across four subscales corresponding with the DSM-5 symptom criteria of PTSD: Intrusion, Avoidance, Negative alterations in cognition and mood, and Alterations in arousal and reactivity. The PCL-5 format used omitted the Criterion A component as participants were already screened for having a service-connected PTSD diagnosis. Rather, current symptom severity was assessed by asking participants to rate their symptomology in relation to a general “stressful experience.” Participants were asked to rate how often each PTSD symptom has affected them in the past month on a scale of 0 (“Not at all”) to 4 (“Extremely”). The scale ranges from 0 to 80, with higher scores indicating greater PTSD symptom severity. Cronbach’s α in the current sample was 0.95 overall with subscale α’s of 0.91 (B), 0.84 (C), 0.88 (D), and 0.86 (E). A total of 31 participants (14%) did not fill out the PCL-5 measure and thus were excluded from analyses that related PTSD symptom severity to outcomes. A total of 11 participants (5%) had missing values, but completed more than 75% of the PCL-5 (*n* = 8 missing one question, *n* = 2 missing two questions, and *n* = 1 missing three questions), allowing for subscale-level mean imputation of missing values.

#### Veteran-Service Dog Closeness

Veterans with a service dog completed the Inclusion of Other in Self (IOS) scale as a measure of their relationship with the service dog. The IOS is a single item, 7-option pictorial scale with demonstrated validity and reliability to measure interpersonal closeness (Aron et al., 1992). The IOS has been previously used as a measure of the human–animal bond (McConnell et al., 2011; LaFollette et al., 2019). The pictorial scale consists of seven diagrams, each with a set of two circles that range from not overlapping (score of 1) to completely overlapping (score of 7). One circle was labeled “you” and the other labeled “service dog.” Participants were asked to “Choose the option that best describes the relationship between you and your service dog.”

#### Importance of Trained and Untrained Service Dog Behaviors

Participants completed a questionnaire quantifying the perceived importance of a list of trained tasks and untrained service dog behaviors (Table 1). The questionnaire was developed with advice from service dog providers and experts in the field of human–animal interaction.

Seven trained tasks were assessed in this study based off tasks trained from the service dog provider (Table 1). These included the dog’s ability to both interrupt and alert to anxiety or distress (including waking from nightmares), as well as positional commands to be used in public such as *block* and *cover*. The *block* command was split into two different variations: block to help provide personal space in public, and block to guard or protect the veteran from others in public. While the physical behavior of the service dog is identical in both versions, the distinction in wording was intentionally chosen to identify differences in veterans’ perceived purpose of the behavior.

Ten untrained behaviors and characteristics were assessed based on qualitative reports from veterans with PTSD service dogs (Taylor et al., 2013; Yount et al., 2013; Krause-Parello and Morales, 2018). These included the service dog’s companionship, non-judgmental support, source of love, calming presence, source of happiness, source of independence, help leaving the house, help connecting to family members, instilling a routine, and help with social interactions.

For each of the seven trained tasks and ten untrained behaviors, participants were asked on a scale of 1 (“Not at all important”) to 5 (“Extremely Important”) how helpful the behavior has been for their PTSD (or how helpful the behavior is *expected to be*, for those on the waitlist). A short narrative description accompanied each task or behavior/characteristic to aid in objectivity in interpretation (Table 1). An overall importance score was calculated for both trained tasks and untrained behaviors by averaging items. Cronbach’s α in the current sample was 0.84 (trained task importance) and 0.87 (untrained behavior importance).

#### Frequency of Trained Task Use

For each of the seven trained tasks, participants were asked how often they currently used each task in a typical day (or how often they *expected to use* each task in a typical day, for those on the waitlist). As this was a free response question, most participants provided numerical frequency values, but text entries were possible. Text entries were coded into numeric responses by the research team (e.g., “Never” or “Once a day” were coded to 0 and 1, respectively, while ranges such as “4–5 times” were coded to 4.5). However, for 20 data points from *n* = 4 participants with a service dog and six data points from *n* = 2 participants from the waitlist, text entries were unable to be coded into a specific numeric value and thus were dropped from analysis (e.g., “all the time” or “only when I’m in public”).

#### PTSD Symptom Specificity of Trained Tasks

Among only participants with a PTSD service dog, participants were given a list of the 20 symptoms from the PCL-5 and asked to indicate the trained tasks that have helped address each symptom using a check all that apply format. Participants were also given the option to indicate “Not Applicable” for any PTSD symptom.

### Analysis Strategy

Analyses were conducted using Statistical Package for the Social Sciences (SPSS 24.0). To compare demographic characteristics by group, independent *t*-tests were conducted for the continuous variable of age and chi-squared tests were conducted for the categorical variables of gender, marital status, and pet dog ownership.

#### Importance of Trained and Untrained Service Dog Behaviors

Prior to analyses, importance values were examined for their distribution which determined a high degree of skewness. Importance values were log-transformed, which corrected the skew to a normal distribution. To compare expected and experienced importance of behaviors, a series of linear regressions were conducted which predicted log-transformed importance from the binary variables of having a service dog or not (yes or no) as well as participant gender (male or female), relationship status (single or married/cohabitating), if there was a pet dog in the home (yes or no), and PTSD severity (total PCL-5 score). Age was also considered as an independent variable, but did not have any significant effects in models (*p*’s > 0.10). Thus, age was excluded from further models to conserve power. Further, to reduce the number of statistical comparisons made, only the average untrained behavior importance score was compared across groups (rather than item-level comparisons). Within-group *t*-tests compared trained task importance to untrained behavior importance.

Linear regressions were conducted to determine the effect of PTSD severity, veteran-service dog relationship, and time since the service dog was placed on log-transformed perceived importance of behaviors. Independent variables included the demographic covariates above and PTSD severity (total PCL-5 score), as well as veteran-service dog closeness (IOS score) and time since service dog placement (in number of months) for those with a service dog. Cohen’s *d* effect sizes were calculated based on the means, standard deviations, and sample sizes of each group using the cutoffs of 0.2 for a small effect, 0.5 for a medium effect, and 0.8 for a large effect (Cohen, 1988).

#### Frequency of Trained Task Use

The distribution of frequency values also had a high degree of skewness with several extreme outliers. To account for the fact that these outliers could lead to significant results that might not be representative, data were winsorized such that extreme values were replaced with the trimmed cutoff of three standard deviations from the mean. Using this approach, a total of 24 extreme values from 10 participants were replaced. After winsorizing, residuals did not follow normality assumptions. Winsorized values were then log-transformed, which resulted in normal residuals in subsequent linear regression models.

#### PTSD Symptom Specificity of Trained Tasks

For each participant, two scores were calculated. First, the number of tasks that were reported to help each PTSD symptom were summed such that a score of 0 indicated that the participant did not perceive any tasks to help the PTSD symptom (and/or they had indicated “Not Applicable”), and a score of 7 indicated that the participant perceived all seven trained tasks as helping the PTSD symptom. An average of this score was taken across all participants to calculate the average number of trained tasks that helped each PTSD symptom, with a possible score range from 0 to 7. Second, the number of PTSD symptoms that were helped by each trained task were summed such that a score of 0 indicated the participant did not perceive the trained task to help any of the listed PTSD symptoms, and a score of 20 indicated the participant perceived the trained task to help all 20 PTSD symptoms. An average of this score was taken across all participants to calculate the mean number of PTSD symptoms helped by each trained task, with a possible score range from 0 to 20. A total of *n* = 10 participants who completed less than half of the PTSD symptom specificity survey were excluded from these summary scores in order to prevent skewed values.

## Results

### Demographics

Participants with a service dog (*n* = 134) and on the waitlist (*n* = 83) did not significantly differ in age (waitlist *M* = 39.63, *SD* = 9.06 years old; service dog *M* = 39.99, *SD* = 8.07 years old; *t* = 0.30, *p* = 0.764; age missing for *n* = 3 individuals with a service dog and *n* = 1 on the waitlist). In addition, groups did not differ by relationship status (waitlist 67% married or cohabitating, service dog 63%; X^2^ = 0.23, *p* = 0.631; relationship status missing for *n* = 2 individuals on the waitlist), or whether they had a pet dog in the home or not (waitlist 45%, service dog 50%; X^2^ = 0.60, *p* = 0.437). However, groups did significantly differ in gender; participants on the waitlist were more likely to be female than those with a service dog (waitlist 66% male, service dog 81% male; X^2^ = 6.59, *p* = 0.010). Groups significantly differed in PTSD symptom severity, with those on the waitlist reporting more severe PTSD symptoms than those with a service dog (waitlist PCL-5 *M* = 58.97, *SD* = 12.96, service dog *M* = 44.34, *SD* = 17.13; *t* = −6.62, *p* < 0.001; Jensen et al., 2020).

### Importance of Trained Tasks and Frequency of Task Use

**FIGURE 1:**
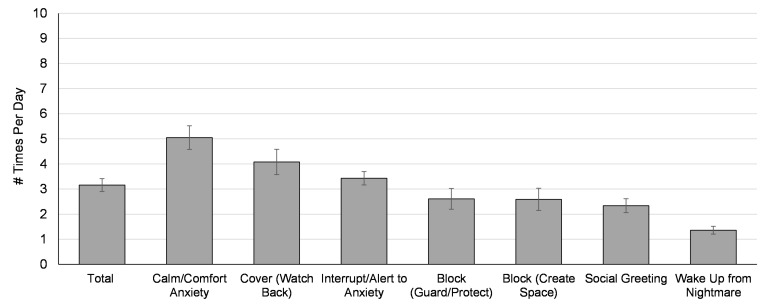
Mean frequency of task use in “a typical day” reported by *n* = 97 veterans with a service dog, ordered from highest to lowest values. Error bars represent the standard error of the mean.

Table 2 displays descriptive statistics of perceived importance and frequency of use of service dog trained tasks. Overall, participants with a service dog reported using a trained task an average of 3.16 (*SD* = 2.54) times a day (Figure 1). Veterans with a service dog rated *calm/comfort to anxiety* as both the most important task and the most frequently used task. Similarly, *cover* and *interrupt/alert to anxiety* were rated as the second and third most important and most frequently used tasks, respectively. *Block to create space* and *block to guard/protect* were rated nearly identically for both importance and frequency. Veterans rated the service dog’s *social greeting* task as the least important behavior for their PTSD and the second least frequently used task. Perceived importance of the *social greeting* task had the largest variance among veterans with a service dog, indicating the most individual variability in responses. The least frequently used service dog task from veterans was *wake up from nightmare*. It is notable that even the lowest-rated tasks were still perceived on average as “moderately” important for veterans’ PTSD. Overall, waitlist expectations of importance and frequency of use of trained tasks tended to be higher than what was experienced among veterans with service dogs (see Results section “Expectations vs. Experiences”).

**TABLE 2:** Means, standard deviations and group comparisons of the expected and experienced importance of trained tasks for PTSD symptoms (1 = Not at all important to 5 = Extremely important) and frequency of trained task use per day.

**Task importance**	**Service dog (*n* = 134)**		**Waitlist (*n* = 83)**		**Group difference**		
	***M***	***SD***	***M***	***SD***	**β**	***p***	***d***
***Total***	3.70	0.82	4.21	0.68	−0.22	0.005**	0.68
Calm/comfort anxiety	4.23	0.97	4.43	0.74	−0.07	0.388	0.23
Interrupt/alert to anxiety	3.98	0.97	4.36	0.79	−0.06	0.447	0.43
Cover (watch back)	3.95	1.13	4.39	0.92	−0.13	0.125	0.43
Block (create space)	3.65	1.14	4.35	0.83	−0.25	0.002**	0.70
Block (guard/protect)	3.63	1.19	4.34	0.85	−0.24	0.002**	0.69
Wake up from nightmare	3.31	1.33	4.06	1.11	−0.18	0.025*	0.61
Social greeting	3.18	1.51	3.54	1.15	−0.21	0.013*	0.27

**Task frequency**	**Service dog (*n* = 97)**		**Waitlist (*n* = 63)**		**Group difference**		
	***M***	***SD***	***M***	***SD***	**β**	***p***	***d***

**Total**	3.16	2.54	5.23	4.08	−0.21	0.019*	0.61
Calm/comfort anxiety	5.05	4.60	6.48	4.84	−0.75	0.407	0.30
Cover (watch back)	4.08	4.90	6.43	6.75	−0.25	0.010*	0.40
Interrupt/alert to anxiety	3.43	2.61	5.92	4.36	−0.24	0.008**	0.69
Block (guard/protect)	2.61	4.02	5.09	5.61	−0.25	0.009**	0.51
Block (create space)	2.59	4.32	5.80	6.98	−0.33	0.001**	0.55
Social greeting	2.34	2.69	3.82	3.52	−0.14	0.214	0.47
Wake up from nightmare	1.36	1.51	2.47	2.22	−0.05	0.621	0.58

Tasks are ordered from highest to lowest mean values within each group. Group difference data includes standardized regression coefficients and p-values controlling for participant gender, relationship status, presence of pet dog in the home, and PTSD severity. Effect sizes are displayed using Cohen’s d; *p < 0.05, **p < 0.01.

### Importance of Untrained Behaviors

Overall, veterans with a service dog rated the importance of untrained behaviors higher than the importance of trained tasks (*M*_trained_ = 3.70 of 5, *M*_untrained_ = 4.42; *t* = −8.50, *p* < 0.001, *d* = 1.04). Table 3 contains descriptive statistics regarding veterans’ perceived importance of untrained service dog behaviors and characteristics. Veterans with a service dog rated all ten untrained behaviors on average as “quite a bit” to “extremely” important for their PTSD symptoms. The most important untrained behavior for helping PTSD symptoms was the dog’s ability to give the veteran something to love and to feel loved in return. The least important untrained behaviors for PTSD were the service dog’s ability to connect them to their family and provide social help in public, but most participants on average indicated these behaviors were still “quite a bit” important for their PTSD. However, connecting to family and social help also had large standard deviations indicating that responses for these characteristics were quite varied. Expected importance of untrained behaviors did not significantly differ from what was experienced by those with a service dog (see Results section “Expectations vs. Experiences”).

**TABLE 3:** Means and standard deviations of the expected and experienced importance for PTSD symptoms (1 = Not at all important to 5 = Extremely important) of untrained service dog behaviors, ordered from highest to lowest values within each group.

**Untrained behavior/characteristics importance**	**Service dog (*n* = 134)**		**Waitlist (*n* = 83)**		**Group difference**		
	***M***	***SD***	***M***	***SD***	**β**	***p***	***d***
**Total**	4.42	0.54	4.41	0.56	0.05	0.534	0.02
Love	4.79	0.52	4.70	0.66			
Companionship	4.75	0.53	4.67	0.57			
Calming	4.67	0.67	4.66	0.65			
Happiness	4.64	0.60	4.55	0.79			
Non-judgmental	4.51	1.13	4.61	0.87			
Routine	4.42	0.75	4.20	0.93			
Independence	4.29	0.85	4.42	0.80			
Leave house	4.29	0.88	4.40	0.90			
Social help	3.95	1.06	3.96	1.10			
Connecting to family	3.92	1.17	3.90	1.11			

**Group difference analysis includes the standardized regression coefficient and p-value controlling for participant gender, relationship status, presence of pet dog in the home, and PTSD severity*.*

### PTSD Symptom Specificity of Trained Tasks

Table 4 contains descriptive statistics regarding the perceived helpfulness of each trained task for individual PTSD symptoms as reported by veterans with a service dog. For each trained task, veterans were asked to indicate which PTSD symptoms they were helpful for (if any). Across the seven trained tasks, there was considerable variability in the number of PTSD symptoms helped. However, the most widely relevant service dog task for veterans’ PTSD symptoms was *calm/comfort to anxiety*, with veterans reporting this task to help an average of 12.73 of the 20 PTSD symptoms. This task was perceived as applicable to symptoms across all four symptom clusters. The second most widely relevant task was *interrupt/alert anxiety*, helping an average of 6.80 of the 20 PTSD symptoms. Most veterans perceived this task as being helpful to several intrusion symptoms as well as symptoms regarding alterations in arousal and reactivity. The task that veterans reported to help the least amount of PTSD symptoms on average was *social greeting*, helping an average of 1.14 PTSD symptoms. *Wake from nightmares* was also reported to help only 1.76 PTSD symptoms on average a majority of veterans reporting this task to help with intrusive dreams.

**TABLE 4:** Means, standard deviations, and population percentages of the PTSD symptom specificity of trained behaviors.

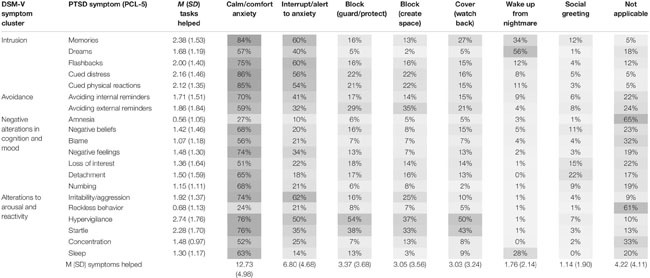

**Percentages represent the proportion of n = 120 veterans with a service dog who indicated that a given task helped each PTSD symptom, with darker colors indicating higher proportions and lighter colors indicating lower proportions. Average tasks helped represents the mean number of tasks (of 7) indicated to help each PTSD symptom by the overall sample. Average symptoms helped represents the mean number of PTSD symptoms (of 20) helped by each trained task for the overall sample. PTSD symptoms are ordered based on the PTSD checklist for DSM-5 (PCL-5), while trained tasks are ordered right to left based on the highest to lowest number of average PTSD symptoms helped*.*

On average, the PTSD symptom helped the most by the service dog’s trained tasks was hypervigilance, with veterans indicating an average of 2.74 trained tasks (of seven) were helpful toward addressing this symptom. Further, 50% or more of veterans reported that four tasks (*interrupt/alert to anxiety*, *calm/comfort to anxiety*, *block to guard/protect*, and/or *cover/watch back*) helped their hypervigilance. Other PTSD symptoms helped by more than two tasks on average included intrusive memories of the traumatic event (*M* = 2.38 tasks), feeling jumpy or easily startled (2.28), feeling distressed when reminded of the traumatic event (2.16), and having strong physical reactions (e.g., heart pounding, and sweating) when reminded of the traumatic event (2.12). On the contrary, the PTSD symptoms that were least helped by the service dog’s trained tasks included trouble remembering the traumatic event (*M* = 0.56 of 7 tasks) and engaging in reckless behavior (*M* = 0.68 tasks). When asked if the service dog’s trained tasks helped these two symptoms, 65 and 61% of veterans, respectively, indicated the service dog’s training was “not applicable” to these symptoms.

### Effect of PTSD Severity, Veteran-Service Dog Closeness, and Time Since Service Dog Placement

Table 5 displays analyses examining the relationships between PTSD severity, veteran-service dog closeness, and time since service dog placement with importance and frequency outcomes. Among veterans with a service dog, there was no effect of PTSD symptom severity on trained task importance, untrained behavior importance, or frequency of task use. Specifically, veterans’ PTSD symptom severity did not predict how often they used trained tasks in a given day, nor how important they rated trained and untrained behaviors for their PTSD. Among veterans with a service dog, veteran-service dog closeness was a stronger predictor of perceived importance and reported frequency of PTSD service dog behaviors (Table 5). Specifically, higher perceived veteran-service dog closeness was associated with higher perceived importance of both trained and untrained behaviors for the veteran’s PTSD. Veterans who reported higher closeness with their service dogs also reported using the service dog’s trained behaviors more often. There was no significant relationship between time since the service dog was placed and perceived trained task importance or untrained behavior importance. However, time since placement was a significant predictor of frequency of task use such that the longer the veteran had the service dog, the less frequently they reported using trained tasks on a daily basis.

**TABLE 5:** Relationship of PTSD severity, veteran-service dog closeness, and time since placement with importance of untrained behaviors and trained tasks for PTSD symptoms and frequency of task use among veterans with service dogs or on the waitlist.

	**Service dog (*n* = 111)**						**Waitlist (*n* = 71)**	
	**PTSD severity**		**Veteran-service dog closeness**		**Time since placement**		**PTSD severity**	
	**β**	***p***	**β**	***p***	**β**	***p***	**β**	***p***
Untrained behavior importance	−0.13	0.157	0.40	<0.001***	−0.03	0.776	0.09	0.437
Trained task importance	−0.04	0.666	0.35	<0.001***	0.03	0.790	0.49	<0.001***
Task frequency	0.15	0.137	0.41	<0.001***	−0.36	0.001**	0.41	0.001**

Data displayed includes standardized regression coefficients and p-values controlling for participant gender, relationship status, and presence of pet dog in home; **p < 0.01, ***p < 0.001.

Among veterans on the waitlist, PTSD symptom severity was a significant predictor of expected importance of trained tasks, but not untrained behaviors (Table 5). That is, veterans on the waitlist with more severe PTSD expected their future service dogs’ trained tasks as being more important for their PTSD than veterans with less severe PTSD symptoms. In addition, PTSD severity was a significant predictor of expected task frequency, such that veterans on the waitlist with more severe PTSD symptoms expected to use the service dog’s trained task more often on a daily basis in the future.

### Expectations vs. Experiences

Overall, waitlist expectations of importance and frequency of use of trained tasks was significantly higher on average than what was experienced among veterans with service dogs. Specifically, after controlling for participant gender, relationship status, presence of a pet dog in the home, and PTSD severity, waitlist participants expected both overall task importance and four of the seven specific trained tasks to be more important for helping their PTSD symptoms than what was experienced by those with a service dog (Table 2). Tasks in which expected importance was not higher than experienced were *calm/comfort anxiety*, *interrupt/alert to anxiety*, and *cover*. Regarding frequency of use, participants on the waitlist again expected to use trained service dog tasks more frequently per day than those with a service dog reported. Specifically, veterans on the waitlist expected to use four of seven trained tasks (*cover*, *interrupt/alert to anxiety*, *block to guard/protect*, *and block to create space*) more frequently than what was reported by those with a service dog (Table 2). Similar to veterans with a service dog, those on the waitlist expected to use *calm/comfort to anxiety* the most often per day, followed by *cover* (*watch back*).

Expected importance of untrained behaviors did not significantly differ from what was experienced by those with a service dog (Table 3). However, both groups reported near-ceiling importance for all 10 untrained behaviors and characteristics. Aligning with experiences from those with a service dog, veterans on the waitlist perceived the service dog’s ability to give the veteran something to love and to feel loved in return as the most important untrained service dog characteristic. Similarly, veterans on the waitlist reported the service dog’s ability to connect them to their family and provide social help in public as the least important untrained behaviors for PTSD. Overall, veterans on the waitlist rated the expected importance of untrained behaviors significantly higher than trained tasks (*M*_untrained_ = 4.41, *M*_trained_ = 4.21; *t* = 2.07, *p* = 0.040, *d* = 0.32).

Among the waitlist, PTSD symptom severity was a significant predictor of expected trained task importance and frequency of task use, but this relationship was not found for veterans with a service dog (Table 5). However, among both groups there was no relationship between PTSD severity and perceptions of the importance of untrained behaviors.

## Discussion

### General

The overall aim of this research was to both document and quantify the therapeutic use of PTSD service dogs to define the intervention while comparing relative expectations of those on the waitlist to everyday experiences of those with a service dog. The specific objectives of this research were to (1) quantify the importance of trained and untrained service dog behaviors toward alleviating PTSD symptoms, (2) quantify how often trained tasks are used while describing their PTSD symptom specificity (2) determine how PTSD symptom severity, the veteran-service dog relationship, and time since the service dog was placed may relate to importance and frequency outcomes, and (3) compare the expectations of those on the waitlist to the everyday experiences of veterans with service dogs. Results from this study offer valuable knowledge toward understanding the specific components and therapeutic value of PTSD service dogs, the PTSD symptoms that are helped most by the service dog’s trained tasks, and quantifying the PTSD service dog intervention among a large and representative sample of military veterans both with a service dog and on the waitlist to receive one.

### Trained Tasks

The first objective served to quantify critical components of the PTSD service dog intervention by describing the perceived importance and frequency of use of the service dog’s trained tasks. Although there was a moderate degree of individual variance observed, results suggest that all seven trained tasks were, in some capacity, valuable aspects of the PTSD service dog intervention from the perspective of this population. Among those with a service dog, all seven tasks were rated on average as “moderately” to “quite a bit” important for veterans’ PTSD. Trained service dog tasks were used on average 3.16 times per day, with individual tasks ranging from an average of 1.36–5.05 times per day. While some trained tasks were broader in their helpfulness toward PTSD symptoms than others, veterans with service dogs reported that all seven trained tasks helped at least one PTSD symptom on average. Results provide critically necessary quantification of the perceived importance, use, and PTSD symptom specificity of psychiatric service dogs’ trained tasks.

The trained tasks of *calm/comfort to anxiety* and *interrupt/alert to anxiety* were among the most centrally valued trained tasks for veterans’ PTSD. These tasks were not only the most important for veterans’ PTSD symptoms, but were also among the most frequently used tasks and rated to help the most number of individual PTSD symptoms. For example, *calm/comfort to anxiety* was reported as the most important task for PTSD (4.23 out of 5), the most frequently used task (5.05 times per day), and the task that helped the most number of specific PTSD symptoms (12.73 out of 20 symptoms on the PTSD Checklist). Similarly, *interrupt/alert to anxiety* was perceived as the second most important task (3.98 out of 5), the third most frequently used task (3.43 times a day) and helped the second most number of specific PTSD symptoms (6.80 out of 20 symptoms). These findings mirror qualitative reports suggesting that these anxiety-reducing service dog behaviors are valued by veterans for reducing hypervigilance and coping with re-experiencing episodes (Vincent et al., 2017a; Yarborough et al., 2017; Bergen-Cico et al., 2018; Crowe et al., 2018; Krause-Parello and Morales, 2018). For example, in a 2017 qualitative study of the benefits of psychiatric service dogs, veterans described how the “nudging” behavior from their service dogs during a flashback episode served to help their PTSD by interrupting the distress, “grounding” the veteran, and reminding the veteran to stay in the present (Yarborough et al., 2018). Previous research with non-PTSD populations has also found that simply having a dog present when experiencing distress reduces both subjective stress (Lass-Hennemann et al., 2014) and objective, physiological biomarkers of stress (Polheber and Matchock, 2013). Overall, findings from this research indicate that the service dog’s ability to respond to the veteran’s distress and serve as a calming presence during anxiety episodes are key mechanistic components of the PTSD service dog intervention.

The *cover* task was the second most frequently used task (4.1 times a day) and was reported to help the PTSD symptoms of hypervigilance and feeling “jumpy” or easily startled. This “watch my back” task is thought to replicate aspects of military comradery in which soldiers will guard each other’s blind spots during combat. Previous qualitative reports have described the value of the *cover* task for reducing hypervigilance in public; veterans describe how their service dogs help “share the burden” of being continuously on alert or aware of approaching people (Yarborough et al., 2018). Interestingly, use of this task had the most variability among participants. Because *cover* is largely encouraged to be used when the veteran is hypervigilant of approaching people (such as in public), the observed variation in the frequency of use may be due to the range of experiences and needs from this population. For example, veterans who frequently engage in public activities may also use the cover task more frequently than a veteran who leaves their house less often. Future research may benefit from examining how veterans use tasks differently in different settings during the trajectory of their recovery and reintegration into society over time.

The *social greeting* task helped an average of 1.14 of 20 PTSD symptoms, thus was less broadly applicable to PTSD symptoms than other trained tasks. However, the task was still rated as “moderately” important on average for participants’ PTSD. Similar to *cover*, the *social greeting* task is trained to especially assist veterans while in public when interacting with other people. Thus, veterans that go out in public more may both use this task more frequently and perceive greater benefit from the task toward alleviating PTSD symptoms such as detachment from others. Research has shown that both pet dogs and service dogs can be useful as a “social bridge” to facilitate social interaction with strangers (e.g., Eddy et al., 1988; McNicholas and Collis, 2000). Additionally, research has found that veterans with PTSD service dogs report less social isolation and more social participation than veterans on the waitlist for a service dog receiving treatment as usual (Bergen-Cico et al., 2018; O’Haire and Rodriguez, 2018; Whitworth et al., 2019). In this context, the *social greeting* task may serve as a key component of this observed improvement in social interactions.

The *wake up from nightmare* task, in which the dog recognizes signs of physical distress in the veteran at night and wakes them from sleep, was also more specific in the PTSD symptoms that were helped. Although this task did not have the breadth of addressing many PTSD symptoms, it had more specificity in targeting PTSD symptoms such as intrusive memories, nightmares, and sleep disturbances. This finding aligns with qualitative reports describing how veterans have benefited from their service dog’s ability to interrupt nightmares and improve sleep quality (Krause-Parello and Morales, 2018; Yarborough et al., 2018). In the current study, 57% of veterans reported that this task helped them with their trauma-related nightmares. It is unknown whether the remaining veterans may have had minimal nightmare symptomology or may have had service dogs that did not actively engage in nightmare-awakening behavior. Regardless, for those veterans that benefit from this trained task, the service dog’s interrupting behavior during nightmares appears to be an important aspect of the PTSD service dog intervention.

Interestingly, neither veterans with a service dog nor on the waitlist rated the two different versions of *block* – *block to create personal space* and *block to guard/protect* – differently in terms of importance, frequency, or value for PTSD symptoms. The *block* task has specifically been subject to controversy; mental health professionals have argued that using block may encourage the veteran to maintain fear and avoidance behaviors in public, which is contradictory to the goals of traditional exposure treatment for PTSD (Kloep et al., 2017). While our research did not specifically quantify this potential relationship, results do suggest that military veterans perceived both versions of *block* to be “moderately” to “quite a bit” important for their PTSD, on average. A second criticism of the *block* task is that its perceived use to guard or protect the veteran from others may perpetuate and reinforce negative views about their environment. While slightly more veterans with a service dog reported *block to guard or protect* as addressing their hypervigilance than *block to create personal space*, frequency of use of either version of the task was not significantly related to the veteran’s current PTSD symptomology. The two versions of *block* may not have been rated differently due to participants not perceiving the nuances of the differential survey wording. For example, some veterans may have perceived *block to guard and protect* as inherently allowing for personal space. In a 2018 qualitative study, veterans described how their service dogs’ stature and presence created a physical barrier between them and others in public to both prevent individuals from coming too close *and* creating a sense of security (Lessard et al., 2018). Future research will be necessary to elucidate the underlying perceptions of veterans who regularly use the *block* task and how it relates to their avoidance symptomology and views regarding their social environment.

### PTSD Symptom Specificity

Among veterans with a service dog, trained tasks addressed almost every PTSD symptom from the DSM-5. On average, intrusion symptoms were helped by the most number of tasks. That is, veterans reported that their service dogs helped mitigate intrusive memories or flashbacks of the traumatic experience as well as internal and physical distress from the memories. These symptoms were mainly addressed by the trained tasks of *calm/comfort to anxiety* and *interrupt/alert to anxiety.* In this context, the service dog’s calming presence and interrupting behaviors (e.g., licking and pawing) can serve to anchor the veteran in the present, thereby distracting them from the flashback while providing a calming sense of relief from the internal and/or external distress.

The two PTSD symptoms that were not helped for a majority of veterans with service dogs were amnesia (i.e., having trouble remembering parts of the traumatic experience) and engaging in risky or reckless behavior. This finding is to be expected since research has suggested that service dogs are not a standalone “cure” for PTSD. Rather, PTSD service dogs are an complementary treatment to address symptoms as a supplement to evidence-based treatment (O’Haire and Rodriguez, 2018). Thus, it is unrealistic to expect a service dog to address all aspects of PTSD symptomology. In a 2017 longitudinal study, veterans’ PTSD symptomology significantly decreased with clinically meaningful change after 3 months with a PTSD service dog, but only 12 of the 17 PCL symptoms showed significant improvement on an item-level (Vincent et al., 2017b). Both this research as well as current findings provide specificity regarding the PTSD symptoms that are both helped and not helped by service dogs. This information is not only critical to guide clinician’s understanding of how these service dogs may benefit PTSD symptomology, but is also important knowledge for service dog providers when educating potential and current clients on how a service dog may help PTSD.

### Untrained Behaviors

Overall, the service dogs’ untrained behaviors were considered more important than trained tasks for veterans’ PTSD. Specifically, among both those with and without a service dog, 8 of the 10 behaviors or characteristics were rated “quite a bit” important for their PTSD (on a scale from “not at all” to “extremely”). These included aspects of the service dog that can also be shared by a pet dog or an emotional support dog such as the dog’s ability to provide companionship, non-judgmental support, love, a calming presence, happiness, and a sense of routine. In a 2013 survey of 30 military veterans with PTSD who benefited from their pet dogs, veterans similarly reported feeling calmer, less lonely, and less depressed from their dog’s companionship (Stern et al., 2013). However, although most veterans reported that their pet dogs tried to “cheer me up when I’m feeling bad,” there was no significant impact of the pet dog on the PTSD symptoms of intrusive memories, flashbacks, or nightmares (Stern et al., 2013). Overall, results from both the Stern et al. (2013) study and the current research suggest that untrained aspects of canine companionship, inherent to most pet dogs, may be therapeutic for the mental and social health of military veterans with PTSD. However, in addition to the helpfulness of the service dog’s specific training toward interrupting and calming anxiety and assisting the veteran in public, this research found that characteristics specific to service dogs (e.g., providing a sense of independence, allowing the veteran to leave the house, and feeling at ease in public) were rated just as highly as the other untrained behaviors such as providing love and companionship. Future research is necessary to fully disentangle how the service’s untrained and trained behaviors may dually contribute to the therapeutic components of the PTSD service dog intervention. Considering the costs and long waitlists associated with preparing and placing trained service dogs, further research is warranted to determine the potential value of pet dogs and emotional support dogs for this population as an alternative.

### Effects of PTSD Severity, Veteran-Service Dog Closeness, and Time Since Placement

Surprisingly, results showed that PTSD severity was not an important significant predictor of task importance or frequency of use among those with a service dog. Specifically, the severity of a veteran’s PTSD did not have a significant relationship with how important the veteran perceived his or her service dog’s trained or untrained behaviors, nor how often he or she used most trained tasks on a daily basis. These null findings may be partially due to the wide variety of experiences from those with a service dog. For example, one might assume that veterans with more severe PTSD both use trained tasks more frequently and view those tasks as more important. However, some veterans with severe PTSD may infrequently leave their house or engage with strangers resulting in less use of tasks that are most suited to being in public, such as the *cover* or *block* tasks. On the other hand, one might assume that veterans with sub-clinical PTSD may use their service dog’s trained tasks less often due to decreased need. However, veterans who are actively reintegrating into society may be using their service dog’s tasks more often to help mitigate symptoms (e.g., in a school or workplace environment). Thus, these individual variances may have diluted any clear relationship on a population level.

Veteran-service dog closeness was a significant predictor of both perceived importance and frequency of use of trained tasks. The closer a veteran perceived their service dog to themselves on the IOS scale, the more they viewed their service dog’s tasks as important for their PTSD and the more frequently they used the tasks. Veteran-service dog closeness was also positively related to the importance of untrained service dog behaviors. These findings confirm the important moderating relationship that the veteran-service dog bond has in explaining PTSD service dog use and benefits. However, the causational direction of this finding is unable to be determined. Specifically, it remains unclear whether obtaining the benefits of a service dog’s trained or untrained behaviors leads to higher perceived closeness, or if veterans with a closer relationship with their dogs perceive their service dog to be more therapeutic for their PTSD. However, it is likely that some of the service dog’s trained tasks such as waking from nightmares or alerting to rising anxiety or distress require a certain degree of closeness between the veteran and service dog to precede frequency. Indeed, qualitative reports have suggested that as the bond grows stronger between the veteran and service dog, the dog becomes more likely to become sensitive to the veteran’s ‘triggers’ and emotional state in order to alert to the veteran’s anxiety, intervene during a flashback, and/or wake him or her from nightmares.

Finally, time since placement of the service dog was a significant predictor of frequency of trained task use. Specifically, veterans who have had their service dogs for longer reported using trained service dog tasks less often than veterans who have had their service dogs for shorter periods. This finding partially supports a popular stance of the PTSD service dog community that reliance on a PTSD service dog decreases over time as the veteran builds healthy coping skills, reintegrates into society, and decreases avoidance behaviors in public. However, our analyses did not take into consideration engagement with other PTSD treatments over time, which may be an important moderating factor of task use. Future, longitudinal research is necessary to fully understand how the use of trained tasks may vary over time and across individual.

### Expectations vs. Experiences

Overall, results suggest that veterans on the waitlist reported higher expectations than what was experienced by those already with a service dog. Specifically, veterans on the waitlist to receive a service dog expected the service dog’s trained tasks to be more important for their PTSD and used more frequently on a daily basis than what was reported by veterans with a service dog. Veterans on the waitlist with more severe PTSD symptoms also expected service dogs’ trained tasks to be more important for their PTSD and to use these tasks more often compared to veterans on the waitlist with less severe PTSD. These findings may be explained partly by veterans’ feelings of hope and excitement regarding their future PTSD service dog, which may not necessarily be a bad thing. In cognitive-behavioral interventions for PTSD and other types of anxiety disorders, this positive motivational state of hope and optimism may actually play a role in treatment success by mediating clinical improvement (Snyder et al., 2000; Gilman et al., 2012).

On the other hand, there is value in education regarding what to expect from a PTSD service dog. While this research did not directly assess veterans’ expectations regarding potentially negative aspects of the service dog intervention, qualitative research with this population has indicated discrepancies between expectations and actual experiences in terms of drawbacks of having a service dog are important. For example, veterans who recently received a service dog report difficulty in coping with the added stressors of maintaining the dog’s training, integrating the dog into their family, and receiving unwanted attention in public (Yarborough et al., 2018). In addition, one crucial expectation is that sometimes improvements in PTSD symptoms and quality of life may not be immediate, and the initial transition period of integrating the PTSD service dog into the veteran’s life may create additional stress, anxiety, and fatigue (Yarborough et al., 2018). In a 2019 survey of PTSD service dog providers, difficulties including discrepancies in the veteran’s and program’s expectations as well as problems with maintaining at-home training were both reported to lead to dropouts (Vincent et al., 2019). In fact, research supports that conducting interventions with patients regarding what to expect from a given treatment can have meaningful effects on improving dropout, satisfaction, and even treatment success (Noble et al., 2001). Regardless of the specific goals and motives that a veteran has for applying for a PTSD service dog, it is important for service dog providers, mental health professionals, and occupational therapists involved in treatment decisions to instill accurate expectations regarding the therapeutic value and potential drawbacks of a PTSD service dog.

### Limitations

This research is not without its limitations. First, the study population was recruited from a single, national service dog provider. We do not know if our findings would be replicated if we had surveyed populations that had received dogs from other PTSD service dog providers. Not only do different providers have varying training philosophies and models (e.g., programs in which the veteran is entirely hands-on in training their service dog), but not all providers train for the same service dog tasks (Vincent et al., 2019). Therefore, future research and replication are necessary to disentangle provider-specific variation in PTSD service dog task use and efficacy. Additionally, the population was limited to military veterans who had experienced service-related trauma. Thus, findings may not generalize to other populations of trauma survivors. Second, a participation bias may have been present such that veterans with a service dog who chose to participate in this research may have had comparatively more positive experiences with their service dogs than those who declined to participate. Veterans who had experienced negative outcomes from obtaining a service dog were also likely not in our participant pool as these individuals often return their service dogs to the provider. As the psychiatric service dog field grows, researchers should begin to quantify both when and why a PTSD service dog may not be efficacious for PTSD symptoms for some individuals. Finally, this research did not aim to quantify past history of stressful life events and specific sources of trauma, but rather assessed currently symptomology via the PCL-5 in relation to a general stressful event. This may have resulted in a mismatch in symptom identification to other current or past sources of trauma. This study also did not quantify other treatments and interventions that veteran participants were engaging in for their PTSD apart from a service dog. However, both trauma type/history and engagement with other PTSD treatments (e.g., medications and psychotherapy) may have important influence on experiences and perceived value of PTSD service dogs. Future research will benefit from examining how these individual differences may explain potential variance in how veterans are incorporating their PTSD service dogs into their lives.

One population limitation is that groups were not equal on all demographic variables; more females were on the waitlist to receive a service dog than already placed with a service dog. The service dog provider schedules separate placement classes for males and females. Thus, this observed difference is likely due to sampling at a time before a large female-only class had occurred. We attempted to control for this limitation by controlling for gender in all models. Participants’ race and ethnicity were also not collected, nor was time spent on the waitlist, which may have explained outcome variance. Another limitation of this research is that it relies on self-reported accounts, so recall or expectancy biases may have been present. However, most of the constructs in this study were subjective experiences in nature, such as the perceived importance of the service dog’s behaviors and perceived closeness with the service dog. Thus, self-reported data was critical to the research question. A final limitation is that we did not exclude veterans who had recently received their service dogs from participation. This decision was made to both maximize sample size and variability in exploring the effects of time since service dog placement on outcomes. Many service dog providers suggest that most veterans require an initial adjustment period of up to 6 months to develop a bond with the service dog and integrate the dog into their routines and lives. Therefore, some variation observed in importance, frequency, and value of trained tasks may have been partially due to the inclusion of veterans who may have still been in this adjustment period. Future longitudinal research will be necessary to determine how the use and perceived value of PTSD service dogs may evolve over the initial time following placement.

### Conclusion

In conclusion, these results provide valuable quantification of the critical components of the PTSD service dog intervention while describing the everyday experiences and expectations surrounding PTSD service dog’s behaviors. This information is critical for advancing our understanding of how and why PTSD service dogs are beneficial for improving PTSD symptomology and quality of life.

The first two objectives of this research documented how important certain service dog behaviors are for a veteran’s PTSD symptoms while quantifying how often trained service dog tasks are used on a daily basis. Findings determined that military veterans with a service dog viewed the dog’s calming and interrupting behaviors when experiencing anxiety as the most important trained tasks for their PTSD, among the most frequently used tasks in a typical day, and the tasks that helped the most number of PTSD symptoms. However, all seven trained service dog tasks were rated as at least “moderately” important for PTSD, used on average at least once per day, and helped almost all of 20 PTSD symptoms except amnesia and reckless behavior. Further, results suggest that the untrained qualities of a PTSD service dog are essential to their therapeutic value; veterans viewed most untrained behaviors and characteristics as “extremely” important for their PTSD, including the dog’s source of love and companionship. Findings provide a much-needed quantification of the clinically relevant value of PTSD service dogs beyond purely qualitative, free-response research.

The second objective of this research aimed to understand how individual differences may contribute to outcomes and change over time. Findings suggest that veteran’s PTSD symptoms did not predict either their perceptions of the importance of their service dog’s behaviors or the use of the service dog’s trained tasks in a typical day. However, veterans who reported feeling closer to their service dogs tended to report using trained tasks more often, and veterans who had their service dogs for longer reported using trained tasks less often. Those reporting more veteran-service dog closeness also viewed the service dog’s trained tasks as more important for their PTSD. Not only are these findings critical within the context of interpreting outcomes in future longitudinal, controlled trials, but they also shed light on the substantial contribution of the human–animal bond in the PTSD service dog intervention.

As a final objective, this research compared expectations of veterans on the waitlist to receive a service dog to the everyday experiences of veterans with a service dog. Findings suggest that, on average, individuals on the waitlist not only expected to use their service dogs more often than what was experienced, but also expected trained tasks to be more important for their PTSD symptoms. Veterans’ PTSD severity also had a significant positive relationship with how important they expected the service dog’s trained tasks to be for their symptoms, in addition to how frequently they expected to use these tasks daily. These findings specifically help to enable providers, practitioners, and veterans to recognize what to expect from service dogs as a complementary treatment for PTSD.

Overall, this study’s findings contribute to emerging knowledge on psychiatric service dogs as a potential complementary treatment option for military veterans with PTSD. This study documented how often trained service dog tasks are used, how important each task is for managing PTSD symptoms, and how these outcomes may relate to PTSD symptom severity, the human–animal bond, and time since receiving the service dog. This research provides critical information to not only interpret research outcomes, but also to optimize future therapeutic efficacy of the PTSD service dog intervention.

## Data Availability Statement

The datasets generated for this study are available on request to the corresponding author.

## Ethics Statement

The studies involving human participants were reviewed and approved by Purdue University Institutional Review Board. The patients/participants provided their voluntary informed consent to participate in this study. The animal study was reviewed and approved by Purdue University Animal Care and Use Committee.

## Author Contributions

KR, ML, NO, and MO’H contributed to the design of the research and development of the online survey. ML and KR contributed to data curation. KR, KH, and MO’H performed the statistical analyses, with KR and KH writing the results section of the manuscript. KR wrote the first draft of the Introduction, Materials and Methods, and Discussion sections of the manuscript. All authors contributed to manuscript revision and editing and approved the final submitted manuscript.

## Conflict of Interest

The authors declare that the research was conducted in the absence of any commercial or financial relationships that could be construed as a potential conflict of interest.
